# Supplementary figures and images for: The total gut mucosal and fecal bacterial load increases in successful treatment of inflammatory bowel disease with infliximab
Source: Microbiol Spectr. 2025 Jul 7;13(8):e01894-24. doi: 10.1128/spectrum.01894-24 (PMC12323318; doi:10.1128/spectrum.01894-24)

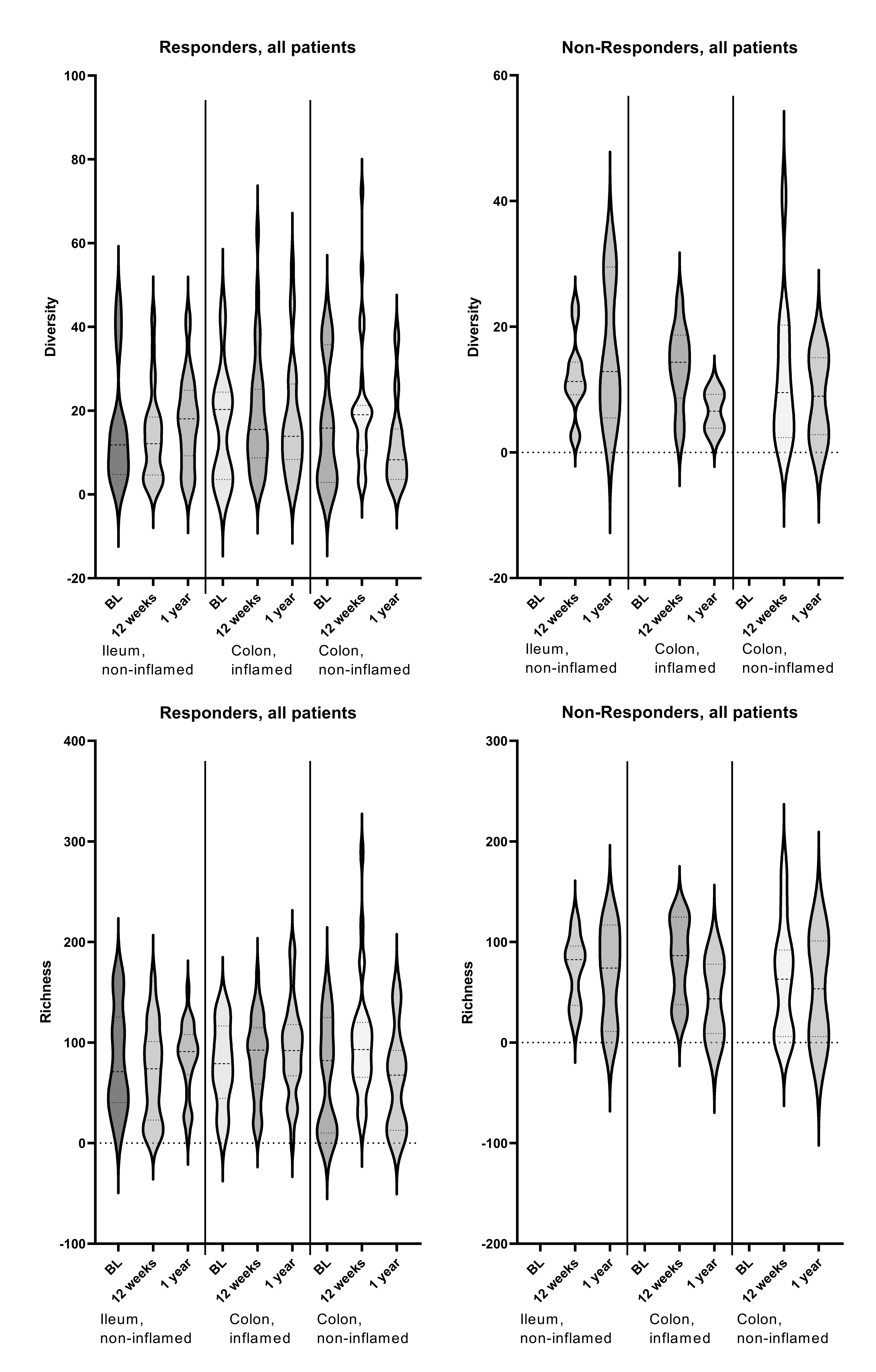

Supplement: Figure S1 — Diversity and richness in mucosal microbiota. [file spectrum.01894-24-s0001.tif]

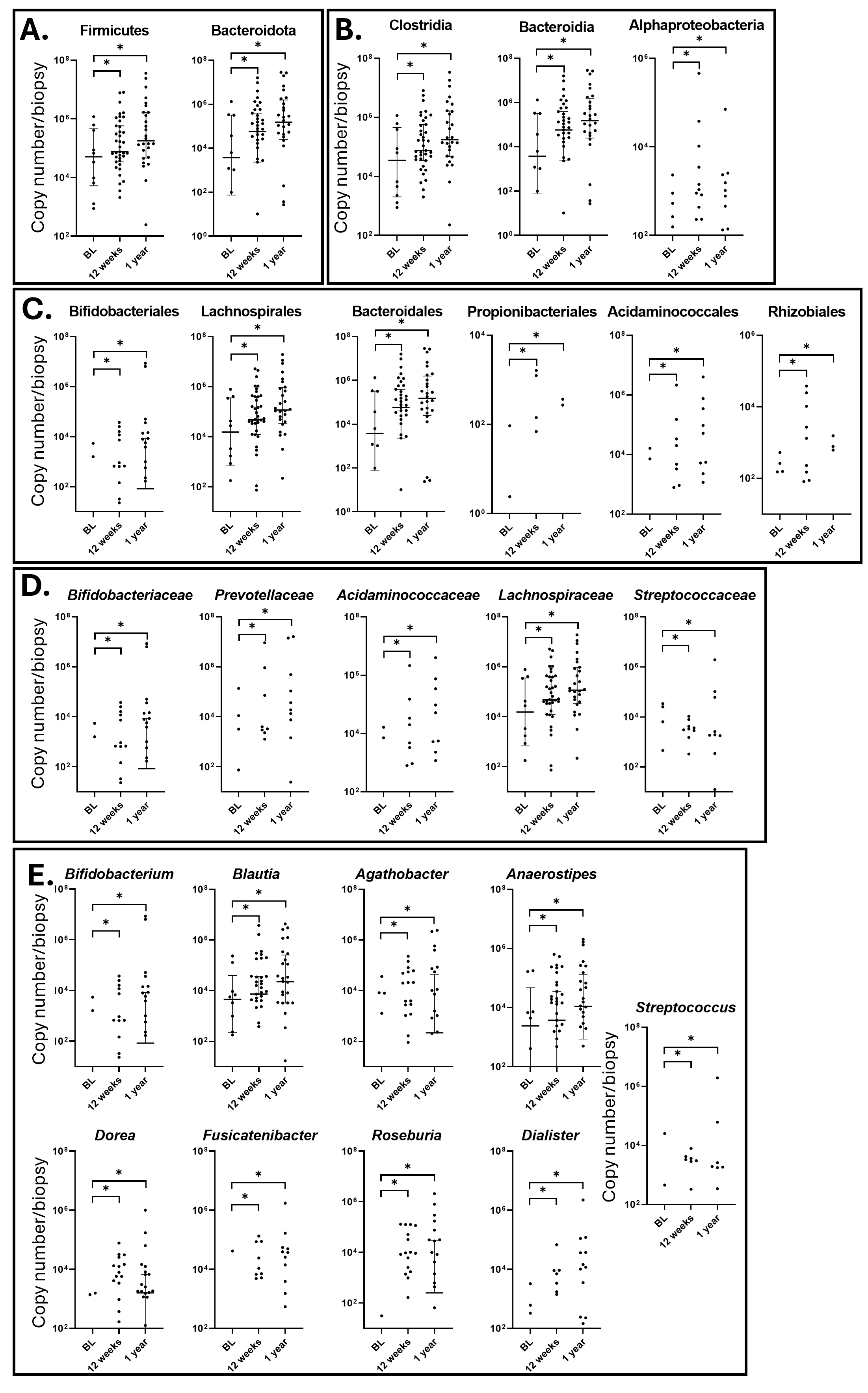

Supplement: Figure S2 — Absolute abundance of bacterial taxonomies in non-inflamed ileum. [file spectrum.01894-24-s0002.tif]

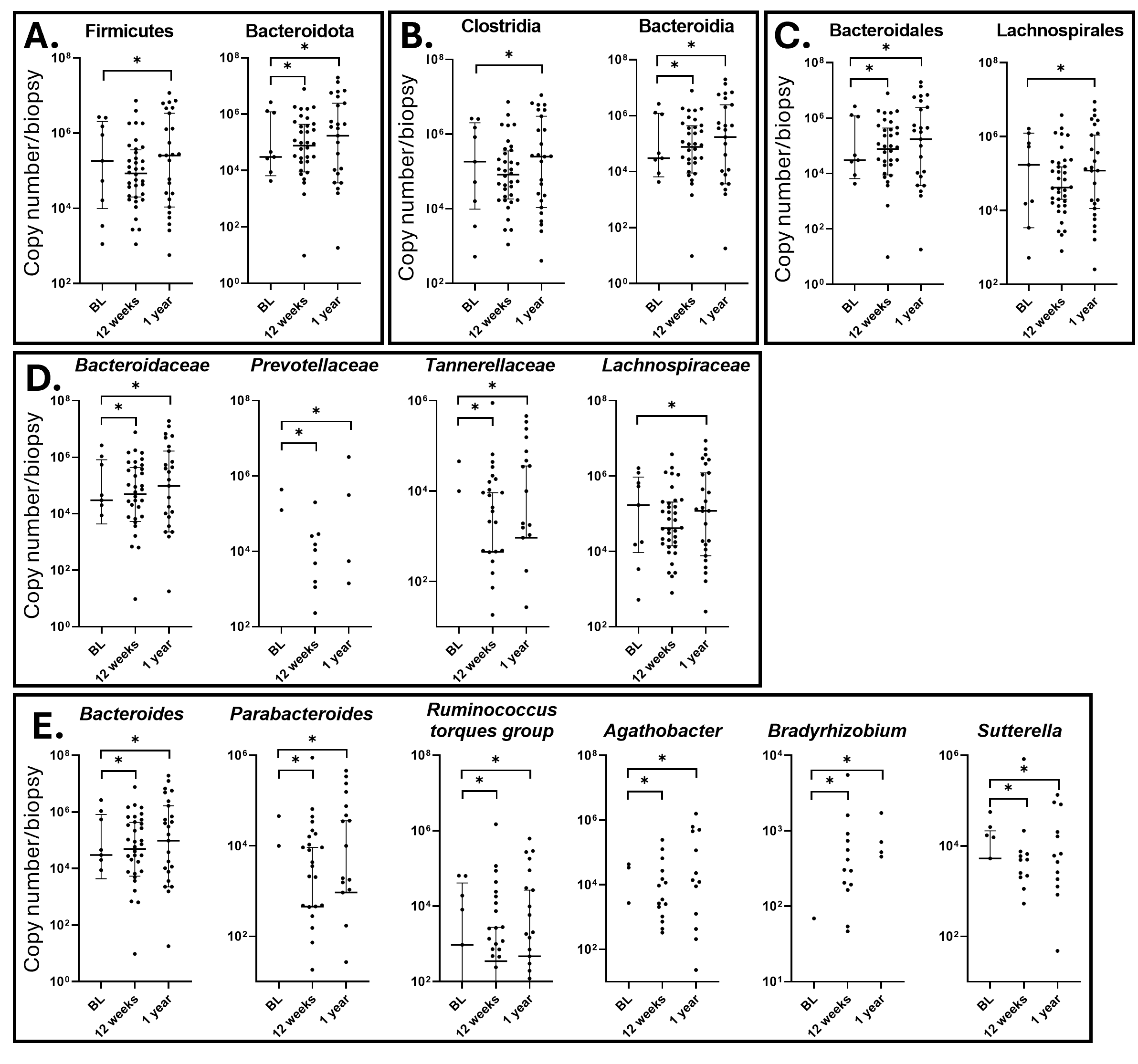

Supplement: Figure S3 — Absolute abundance of bacterial taxonomies in non-inflamed colon. [file spectrum.01894-24-s0003.tif]

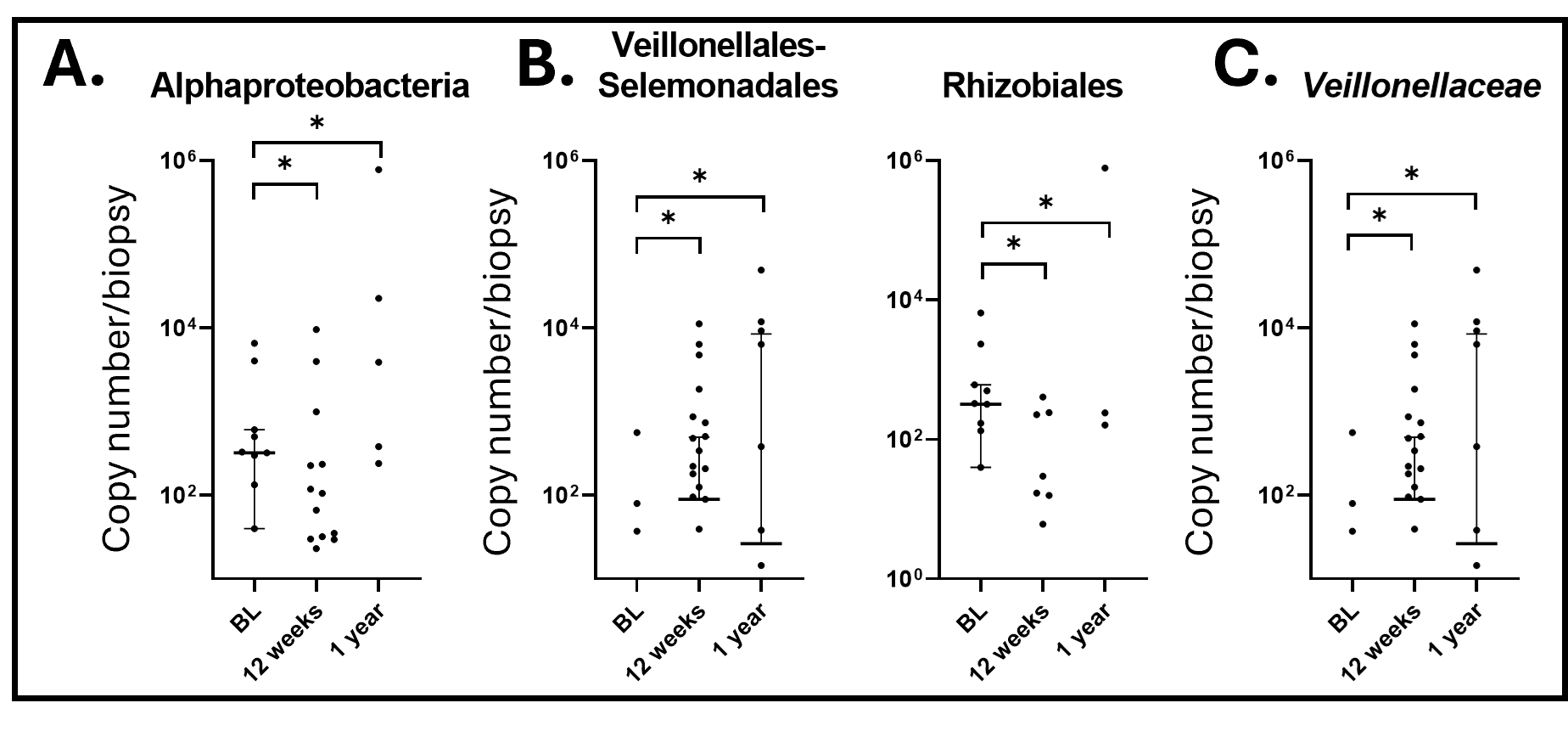

Supplement: Figure S4 — Absolute abundance of bacterial taxonomies in inflamed colon. [file spectrum.01894-24-s0004.tif]

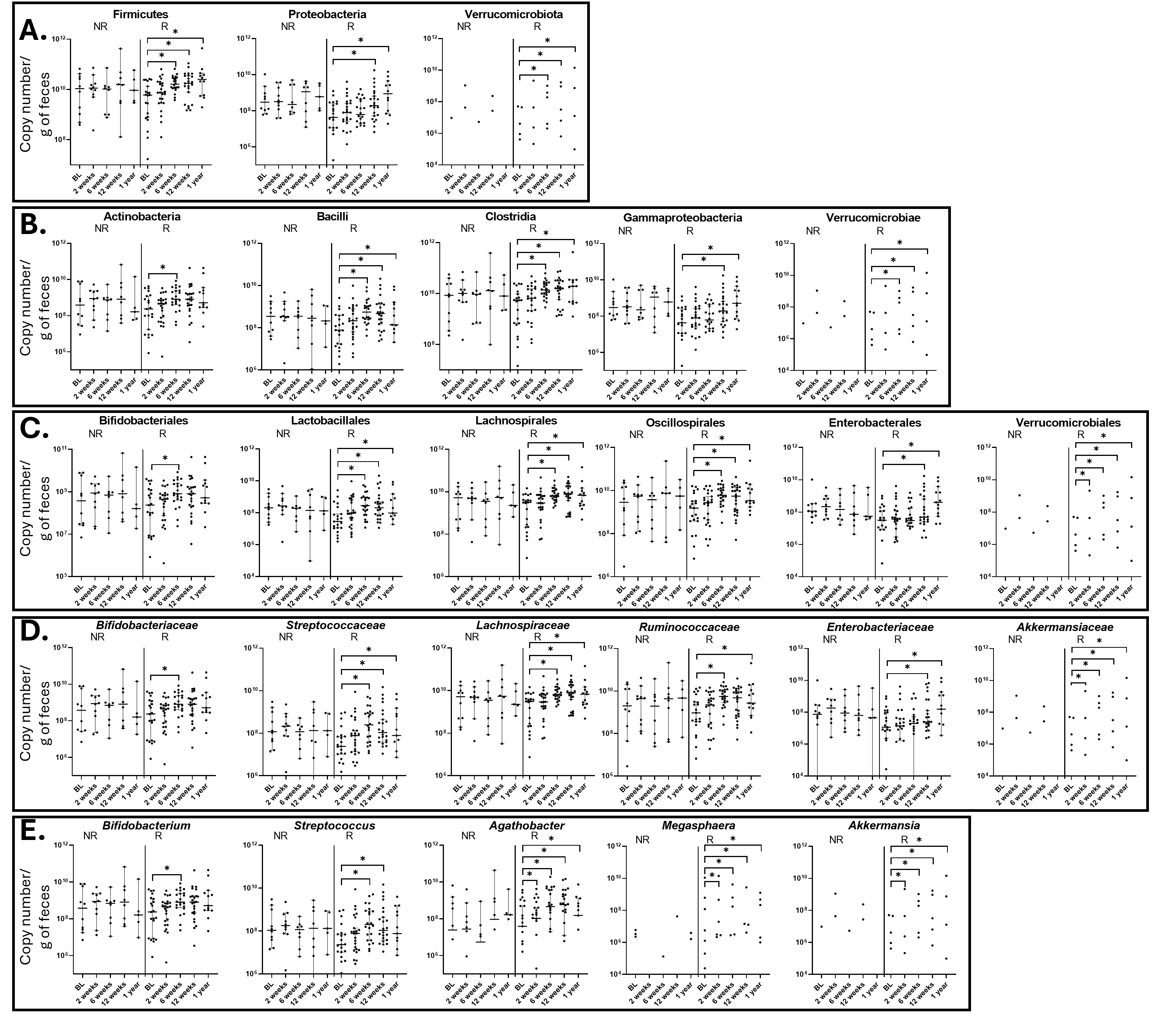

Supplement: Figure S5 — Absolute abundance of bacterial taxonomies in fecal samples of Crohn's disease patients. [file spectrum.01894-24-s0005.tif]

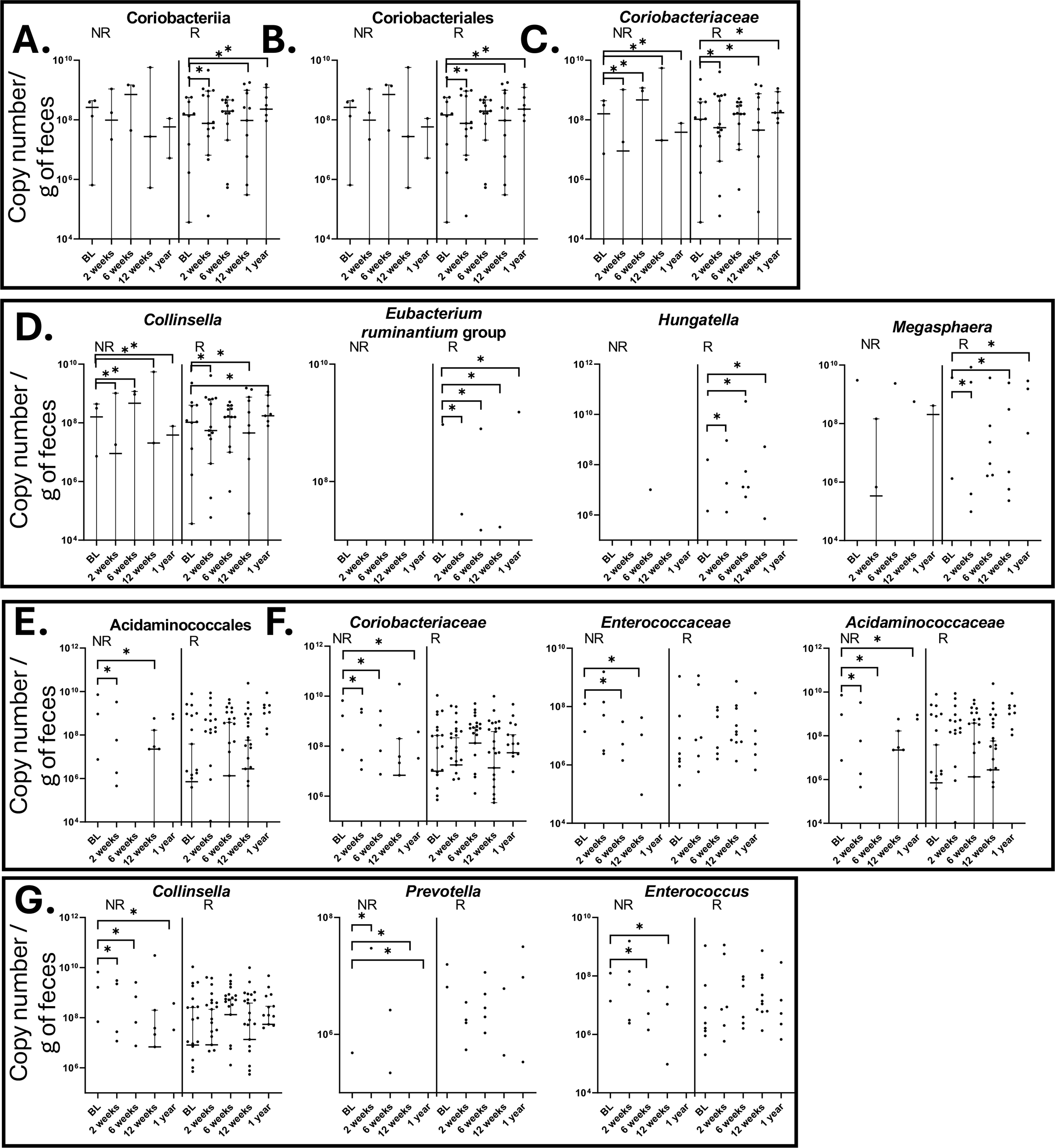

Supplement: Figure S6 — The absolute abundance of bacterial taxonomies that significantly differed in fecal samples of Crohn’s disease responders to infliximab and in fecal samples of ulcerative colitis non-responders to infliximab. [file spectrum.01894-24-s0006.tif]
