# Supplementary material for: The total gut mucosal and fecal bacterial load increases in successful treatment of inflammatory bowel disease with infliximab
Source: Microbiol Spectr. 2025 Jul 7;13(8):e01894-24. doi: 10.1128/spectrum.01894-24 (PMC12323318; doi:10.1128/spectrum.01894-24)
Supplement: Supplemental legends — Legends for Fig. S1 to S6. [file spectrum.01894-24-s0007.docx]

**Supplementary Figure legends**:

**Supplementary Figure S1**. Diversity and richness in non-inflamed ileum, inflamed colon, and non-inflamed colon samples stratified by response to infliximab. BL = baseline, before start of infliximab treatment.

**Supplementary Figure S2**. The absolute abundance of bacterial taxonomies that significantly differed between the time points in **non-inflamed ileum** samples of **responders** to infliximab (including **all patients**) at A. phylum, B. class, C. order, D. family and E. genus level.

**Supplementary Figure S3**. The absolute abundance of bacterial taxonomies that significantly differed between the time points in **non-inflamed colon** samples of **responders** to infliximab (including **all patients**) at A. phylum, B. class, C. order, D. family and E. genus level.

**Supplementary Figure S4**. The absolute abundance of bacterial taxonomies that significantly differed between the time points in **inflamed colon** samples of **responders** to infliximab (including **all patients**) at A. class, B. order and C. family level.

**Supplementary Figure S5.** The absolute abundance of bacterial taxonomies that significantly differed between the time points in **fecal** samples of **ulcerative colitis** responder to infliximab at A. phylum, B. class, C. order, D. family and E. genus level.

**Supplementary Figure S6**. The absolute abundance of bacterial taxonomies that significantly differed between the time points in **fecal** samples of **Crohn’s disease responders** to infliximab at A. class, B. order, C. family, and D. genus level as well as in fecal samples of **ulcerative colitis non-responders** to infliximab at E. order, F. family, and G. genus level.
